# Standardized multi-planar reformation improves the reliability of the assessment of the anterolateral ligament in ACL-deficient knees

**DOI:** 10.1007/s00167-023-07343-w

**Published:** 2023-02-23

**Authors:** Silvan Hess, Andreas Hecker, Rainer J. Egli, Sophie C. Eberlein, Frank M. Klenke

**Affiliations:** 1 Department of Orthopaedic Surgery and Traumatology, Inselspital, Bern University Hospital, University of Bern, Freiburgstrasse 18, 3010 Bern, Switzerland; 2 Department of Diagnostic, Interventional and Pediatric Radiology, Inselspital, Bern University Hospital, University of Bern, Bern, Switzerland

**Keywords:** ALL, ACL, MRI, Multi-planar reformation, Knee, Sport

## Abstract

**Purpose:**

The anterolateral ligament (ALL) is an important structure for controlling anterolateral rotatory stability of the knee. Its assessment, however, is difficult using standardized MRI images. The goal of this study was to assess the reliability of judging the integrity of the ALL on multi-planar reformatted (MPR) MRI images and on standard coronal reformatted (SCR) MRI images in knees with an anterior cruciate ligament (ACL) rupture.

**Methods:**

Forty-eight patients (14 females, 34 males, 30 ± 6 years (mean age ± standard deviation)) with acute ACL ruptures (< 2 weeks) and no additional knee injuries (except segond fractures) were included. Images were assessed by two independent raters twice with at least a 2-week interval in between. The assessment was first performed on SCR images and thereafter on MPR images. Images were judged for assessability of the ALL and then the integrity of the ALL was rated.

**Results:**

Depending on rater and read, the ALL was judged as “torn” in between 5 (10.4%) and 11 (22.9%) patients out of 48 patients on SCR images. On MRP images, the ALL was judged as “torn” in between 5 (10.4%) and 6 (12.5%) patients out of 48 patients, depending on rater and read. Inter- and intra-rater reliability for the assessment of the ALL using MPR images was “substantial” to “almost perfect”. Inter- and intra-rater reliability for the assessment using SCR was “fair” to “substantial”.

**Conclusion:**

MPR images should be used when assessing the integrity of the ALL. Assessment quality is independent of patient positioning during MRI acquisition and the ALL can be displayed in full length on one image.

**Level of evidence:**

Level III

## Introduction

Over the last decade, the anterolateral aspect of the knee has been investigated thoroughly and found to be important for restraining “anterolateral rotatory instability” of the knee [1]. Among the structures of the “anterolateral complex”, the anterolateral ligament (ALL) has gained the most attention. Increased rotational laxity in anterior cruciate ligament (ACL) and ALL-deficient knees compared to knees with an isolated ACL deficiency has been reported by biomechanical and clinical studies [2–4]. The most compelling evidence is thereby presented by two recent studies, one large randomized controlled trial [2] and one retrospective matched-pair cohort study [5], which found a clinically relevant reduction in ACL graft ruptures if an additional lateral extra-articular tenodesis (LET) [2] or an ALL reconstruction [5] was performed in knees with an ACL rupture and suspected concomitant ALL rupture. While the ALL is recognized as an important structure and surgical techniques for its reconstruction are available, indications are less clear. Current guidelines recommend a LET procedure in ACL-deficient knees if a mixture of clinical findings, patient characteristics and/or specific features on MRI images are present. Yet, assessment of the ALL on standard MRI images is challenging. A recent systematic review found inter- and intra-rater reliability ranging from 0.04 to 1.0 and 0.14 to 1.0, respectively [6]. These authors further concluded that the entire ligament was rarely visible [7, 8]. Furthermore, there is evidence that MRI may lack sensitivity because injury rates reported from open surgical exploration in ACL-deficient knees are higher than those reported by MRI studies [9]. Some recent guidelines thus questioned the value of MRI findings as criteria for or against a LET [10].

Multi-planar reformation (MPR) might be a way to improve the visibility of the ALL and improve reliability in judging its integrity. Studies reported significantly higher visualization rates with the use of this technique in healthy knees [11, 12]. Muramatsu et al. further reported higher rates of ALL injuries in acutely ACL-injured knees with this technique compared to rates reported in the literature based on standard MRI images, thus supporting the lack of sensitivity of the standard MRI images [13]. The goal of this study was to apply a previously described MPR technique (which aligns the reference plane directly to the course of the ALL) to knees with an acute ACL rupture and assess the reliability of judging the integrity of the ALL [11]. A higher reliability using MRP compared to SCR could improve the decision-making for/or against a LET/ALL reconstruction and thereby improve outcomes after ACL reconstruction. The following hypothesis was assessed:

- The reliability of the ALL assessment is higher using MRP MRI images compared to standard coronal reformatted (SCR) MRI images.

## Materials and methods

The study was approved by the local ethical committee (Cantonal Research Ethics Commission, Bern, Switzerland, 2020-01559). The ethics committee waived the need to obtain informed consent in this study, according to Article 34 of the Swiss human research act. All procedures performed were in accordance with the ethical standards of the institutional and/or national research committee and with the 1964 Declaration of Helsinki and its later amendments or comparable ethical standards.

For this retrospective cohort study, the hospital’s picture archiving and communication system (PACS) was searched for patients meeting the following inclusion criteria:

(I) 3 T MRI showing an ACL rupture after acute injury between January 2018 and December 2019
(II) Time between trauma and MRI images less than 2 weeks
(III) Age between 20 and 40 years at time of imaging
(IV) Availability of a 3-dimensional intermediate weighted (proton weighted) fat-suppressed sequence (3D PD VISTA SPAIR; TR 1300 ms, TE 32 ms, slice thickness 0.7 mm) and a standard coronal reformation (slice thickness 1 mm, reformation directly performed at the MRI console).

This search resulted in 642 patients, which were then screened for the following exclusion criteria: (I) any additional knee injuries (meniscus, ligament, cartilage), (II) previous surgery around the knee. Forty-eight patients (14 females, 34 males) with a mean age (± standard deviation (SD) of 30 ± 6 years could be included in the study.

Images were assessed by two independent observers using Sectra IDS7 PACS (Sectra medical, Linköping, Sweden). Observer 1 was a fellowship-trained orthopaedic surgeon (HA) specialized in knee surgery and observer 2 was a radiologist with fellowship training in musculoskeletal radiology (RJE). Both observers performed measurements twice individually with at least 2-week interval between the reads. Both raters were blinded to the treatment and patient history.

The assessment was first performed on SCR and thereafter on MPR images. The latter was created using the method described by Hecker et al. [11]: first, the axial and coronal planes were aligned to the distal and posterior edges of the femoral condyles, respectively. Next, the centre of the MPR coordinate system was shifted to the proximal and posterior edge of the lateral epicondyle. Then, the coronal plane was tilted on the sagittal images until a continuous structure could be identified from the lateral epicondyle to a point on the tibia halfway between Gerdy’s tubercle and the tip of the fibular head. Hecker et al. reported full-length visualization of the ALL in 87% to 98% of all patients in a healthy population (*n* = 47) using this MPR compared to 26% to 49% with SCR [11].

**Fig. 1:**
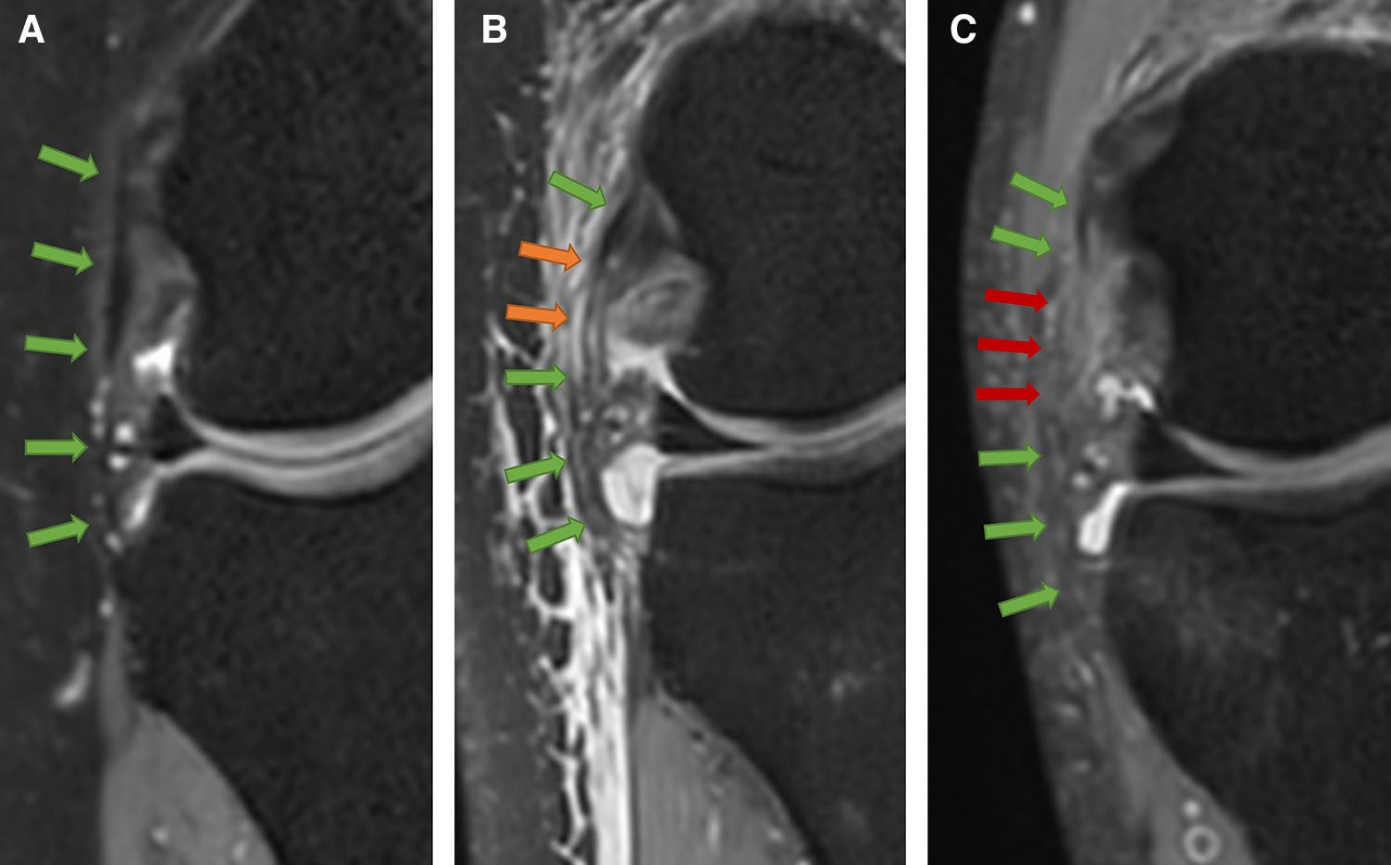
An example of each category proposed by Muramatsu et al. based on MPR MRI images. The ALL was rated as **A** “intact” if a continuous, clearly defined low-signal band was visible, or as **B** “strained” if “warping”, “thinning”, or “iso-signal changes” was visible or as **C** “torn” if no clear continuity was visible. The intact part of the ALL is indicated by the green arrows, the strained part by the orange arrows and the torn part by red arrows

Images were first judged for assessability on SCR, whereby images were rated as “not assessable” if the ALL could not be visualized in total. Regardless of this judgement, raters subsequently assessed the ALL for integrity using the definition proposed by Muramatsu et al. [13]. The ALL was rated as “intact” if a continuous, clearly defined low-signal band was visible; rated as “strained” if showing “warping”, “thinning”, or “iso-signal changes” and as “torn” if no clear continuity was visible. Figure 1 shows an example for each rating based on MRP images. In addition, raters were again offered the additional option to choose “not assessable”, in case they did not feel able to reliability judge the integrity of the ligament. In addition, results were transformed as follows: the ALL was considered “intact” if rated as “intact” or “strained”. The pooling was performed to take into account the clinical consequence of the rating, whereby a “strained” ALL might not need to be addressed surgically.

### Statistical analysis

Statistical analysis was performed using R Statistical Software (R version 4.1., Foundation for Statistical Computing, Vienna, Austria) with “irr” and “irrCAC” packages. Descriptive statistics, such as means, ranges, and measures of variance (e.g. SD), 95% confidence interval (CI)) are presented for normally distributed data. A power analysis was performed aiming to be able to detect an improvement in the agreement of 30% with a power of 0.85 with a two-sided alpha of 0.05, which resulted in a needed sample size of 39. Inter- and intra-rater reliability for categorical data is usually assessed using the method proposed by Cohen (known as “Cohen’s Kappa”) [14]. However, Cohen’s method has the disadvantage of resulting in low values if the prevalence of one category is much lower than the other. Gwet’s agreement coefficient (Gwet’s AC1) is less affected by prevalence and data distribution and was, therefore, used for the analysis. The scale proposed by Landis and Koch for interpretation of the results was used, whereby a value of 0.01–0.2 is considered a “slight”, a value between 0.21 and 0.4 a “fair”, a value between 0.41 and 0.6 a “moderate”, a value between 0.61 and 0.8 a “substantial” and a value above 0.81 as “almost perfect” agreement [15]. Furthermore, the inter-rater agreement for each rating was compared between the two methods (SCR images and MRP images) using McNemar-test.

## Results

Depending on the rater and read, the ALL was classified as “torn” (Type C) in between 5 (10.4%) and 11 (22.9%) patients out of 48 patients on SCR images. On MRP images, the ALL was classified as “torn” (Type C) in between 5 (10.4%) and 6 (12.5%) patients out of 48 patients, depending on the rater and read. Table 1 shows the reported prevalence of each category separated by read (first, second) and rater (A, B). Table 2 shows the inter- and intra-rater agreement coefficients and their interpretation. Table 3 shows the inter- and intra-rater agreement coefficients and their interpretation after transformation. Based on non-transformed data, inter-rater agreement was significantly higher using MRP images in the first rate (*p* = 0.01), but similar in the second rate (n.s.). Based on the transformed data, inter-rater agreement was significantly higher using MRP images (*p* < 0.000 for the first rate, *p* = 0.01 for the second rate).

**Table 1:** Prevalence of each category (Type A, B, C injury) subdivided by rater (A, B) and read (first, second)

	Rater A				Rater B			
	Read 1		Read 2		Read 1		Read 2	
	*N*	%	*N*	%	*N*	%	*N*	%
Assessability on SCR								
No	34	69.4	34	69.4	26	53.1	27	55.1
Yes	14	28.6	14	28.6	22	44.9	21	42.9
Rating coronal								
Not assessable	2	4.1	0	0.0	3	6.1	5	10.2
Type A	8	16.3	11	22.4	9	18.4	9	18.4
Type B	30	61.2	26	53.1	29	59.2	29	59.2
Type C	8	16.3	11	22.4	7	14.3	5	10.2
Rating MPR								
Not assessable	1	2.0	0	0.0	1	2.0	0	0.0
Type A	11	22.4	13	26.5	11	22.4	9	18.4
Type B	31	63.3	29	59.2	31	63.3	35	71.4
Type C	5	10.2	6	12.2	5	10.2	4	8.2

*Type A* continuous, clearly defined low-signal band, *Type B* warping, thinning, or iso-signal changes, *Type C* without clear continuity. *MPR* multi-planar reformation, *Coronal* standard coronal reformation (SCR)

**Table 2:** Inter- and intra-rater reliability of the assessment of the ALL using SCR and MPR images

Inter-rater reliability						
	Read 1			Read 2		
	AC1	*p*-value	Interpretation	AC1	*p*-value	Interpretation
Assessability on SCR	0.373	< 0.05	Fair	0.262	n.s	Fair
Rating coronal	0.461	< 0.05	Moderate	0.376	< 0.05	Fair
Rating MPR	0.849	< 0.05	Almost perfect	0.503	< 0.05	Moderate

Intra-rater reliability						
	Rater 1			Rater 2		
	AC1	*p*-value	Interpretation	AC1	*p*-value	Interpretation
Assessability on SCR	0.36	< 0.05	Fair	0.46	< 0.05	Moderate
Rating coronal	0.72	< 0.05	Substantial	0.66	< 0.05	Substantial
Rating MPR	0.77	< 0.05	Substantial	0.70	< 0.05	Substantial

*MPR* multi-planar reformation, *Coronal* standard coronal reformation (SCR). *AC1* Gwet’s AC1, *Interpretation* Landis and Koch’s interpretation for inter- and intra-rater reliability

**Table 3:** Inter- and intra-rater reliability of the assessment of the ALL using SCR and MPR images after transformation

Inter-rater reliability						
	Read 1			Read 2		
	AC1	*p*-value	Interpretation	AC1	*p*-value	Interpretation
Rating coronal	0.67	< 0.05	Substantial	0.57	< 0.05	Moderate
Rating MPR	0.95	< 0.05	Almost perfect	0.90	< 0.05	Almost perfect

Intra-rater reliability						
	Rater 1			Rater 2		
	AC1	*p*-value	Interpretation	AC1	*p*-value	Interpretation
Rating coronal	0.827	< 0.05	Almost perfect	0.803	< 0.05	Almost perfect
Rating MPR	0.954	< 0.05	Almost perfect	0.910	< 0.05	Almost perfect

*MPR* multi-planar reformation, *Coronal* standard coronal reformation (SCR). *AC1* Gwet’s AC1, *Interpretation* Landis and Koch’s interpretation for inter- and intra-rater reliability

## Discussion

The most important findings of the present study were that the ALL can reliably be assessed using MPR images and that the reliability of its assessment is higher with MPR images than with SCR images. Surgeons treating patients with acute ACL rupture are faced with the question if isolated ACL reconstruction is sufficient or if additional procedures are needed. MRI assessment of the anterolateral structures of the knee thereby might help to identify patients benefiting from an additional lateral procedure. The first step in this assessment is the identification of the ALL, which is difficult because the ALL can often not be visualized in full length using standard MRI images [6, 11, 13, 16]. However, identification of the ALL is improved when MRP images are used instead of SRC images based on our results. The ALL was more often judged as assessable on MPR images compared to SCR images and the inter- as well as intra-rater reliability improved with MPR images. Similar findings were reported by others, who assessed the ALL in healthy [11, 12] and ACL-deficient knees using MPR images [13, 17]. Klontzas et al. assessed the ALL in 14 healthy volunteers on coronal and sagittal 2D MRI images from both a 1.5- and 3-Tesla scanner as well as on MPR images based on 3D gradient echo constructive interference in steady-state sequences [12]. Their findings illustrate the problems associated with identifying the ALL using standard MRI images. In their study, a ligamentous structure possibly representing the ALL was confirmed on coronal images in 90% of knees (on images from both scanners), yet the same structure could not be visualized in any of the corresponding sagittal images. On MPR images, on the other hand, the ALL could distinctly be visualized in 2 planes in 24 of 26 knees (92.3%). However, the technique used in this study might have some advantages compared to the previously reported techniques [12, 13]. Most studies orientated their reference plane according to anatomical landmarks. In the present study, anatomical landmarks were only used as starting point and the reference plane was oriented exactly to the course of the ALL with the aim to display the full length of the ALL on one image.

**Fig. 2:**
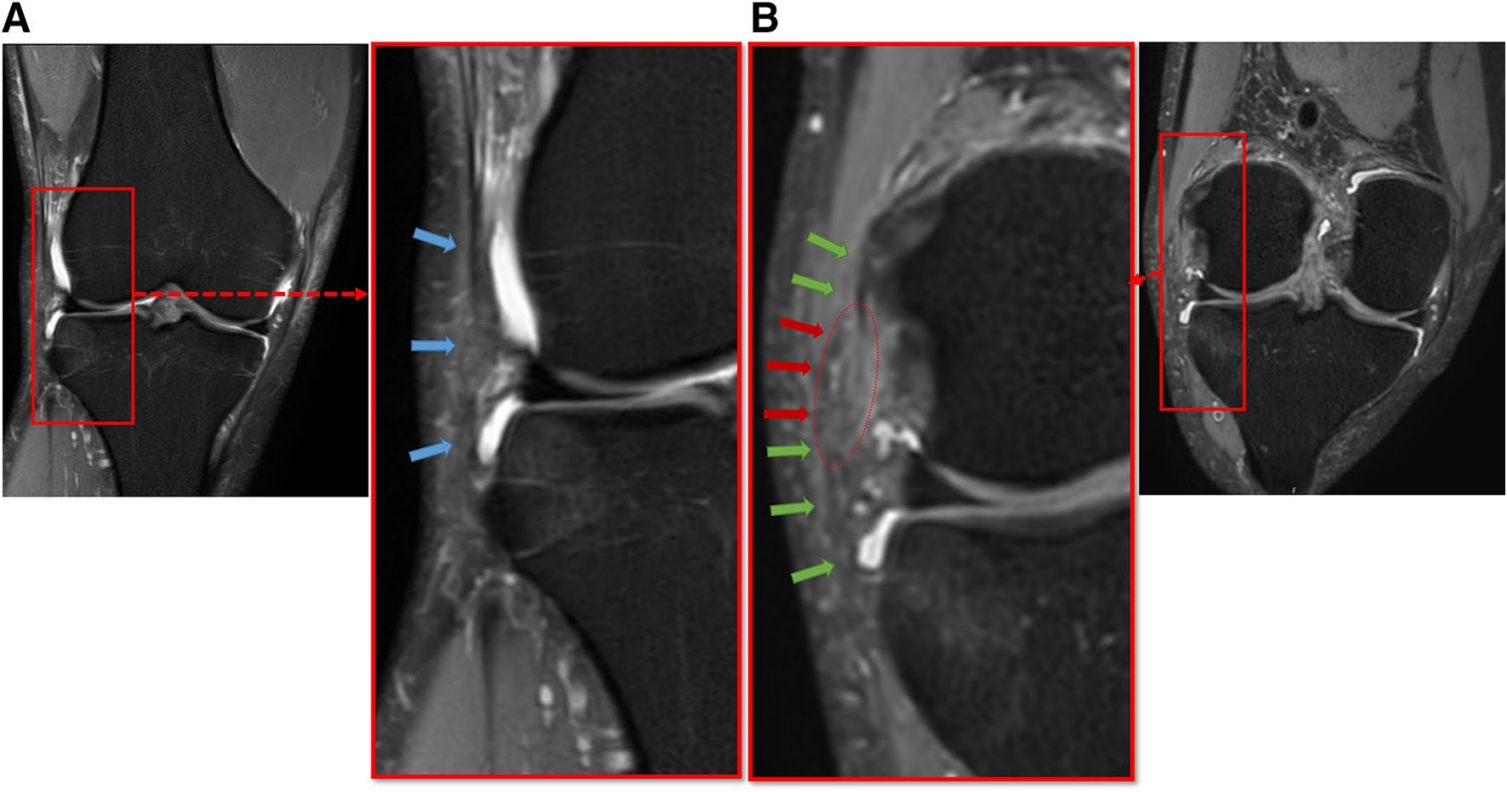
An example demonstrating the advantages of MPR MRI images. The ALL cannot be distinctly visualized as a continuous structure from its origin on the lateral femora to its attachment on the lateral tibia based on SCR images (**A**). Judgement based on these SCR images is difficult and raters judged the ALL as “strained”. However, it is clearly visible based on the MPR images (**B**) and raters judged it as torn. On the right, the red arrows indicated the torn part of ALL, green arrows indicate the remnants of the ALL. On the left, the blue arrows indicate the suspected ALL on the SRC image

The second step in the assessment of the ALL includes the reader’s judgement of the ALL’s integrity, which is challenging because clear definitions for a torn or insufficient ALL are lacking. Previous studies rated the ALL as “torn”, “sprained”, “partially ruptured” or “incompletely injured” using various criteria for each category [6]. There is only one study comparing MRI imaging findings and open exploration of the ALL, whereby significantly higher rates of injuries to the anterolateral capsule were found during open exploration [18]. However, SRC images were used instead of MPR images and the ALL and the anterolateral capsule were considered a single unit, which limits the generalization of their findings. More recently, MRI findings, ultrasound findings, arthroscopic findings and clinical findings of patients with an isolated ACL injury were compared by Rauer et al. [19]. Significantly lower rates of injuries to the anterolateral capsule during arthroscopy compared to rates based on MRI and ultrasound were found. The sensitivity or specificity of any of the published MRI assessments on the ALL is therefore unknown and imaging findings must thus always be interpreted with regard to the clinical findings. However, distinguishing between a “strained” and a “intact” ALL might not be necessary because only a torn ALL with the corresponding clinic may be considered an indication for an additional surgical procedure. Hence, an additional analysis merging all “strained” and “intact” ALL rates was performed, which resulted in a higher inter- and intra-rater reliability. It could thus be argued that a clinically relevant rupture of the ALL can reliable be detected using MPR MRI images. The benefits of using MPR images and our technique in day-by-day clinic work are shown in Fig. 2. Decision-making for or against a LET/ALL reconstruction could be improved using MRP images since the assessment is more consistent.

There are only two studies describing the natural history of the ALL after acute ACL rupture and ACL repair [17, 20]. Both reported no healing potential in patients with a full-thickness ALL tear and limited healing potential in patients with a partial ALL tear (10 out of 27 (37%) patients with partial-ruptured/strained ALLs showed complete healing after 12 months). However, only 4 (17%) out of 23 patients [17] and 4 (1.5%) out of 38 of the patients [20] with a “non-fully healed” or poorly healed ALL had a positive pivot shift test, questioning the ability of MRI images to judge the healing status of the ALL. Further studies are needed to assess the healing potential of the ALL and the clinical consequences of a “strained” or “non-fully healed” ALL. Yet, in patients with a full-thickness ALL tear, surgeons should be aware of the very limited healing potential of the ALL.

This study has several limitations. First and foremost, MRI findings could not be compared to clinical findings because no clinical data were available. Although clinical data could add additional value to the study, it is not necessary in order to assess the reliability of the ALL’s assessment on MRI images. Second, the true sensitivity or specificity of our MRI assessments is unknown since no gold standard (e.g. open exploration) was available for comparison. As mentioned above, this limitation affects almost all previous studies and MRI findings must always be interpreted with regard to clinical findings. Third, the study cohort had an unequal gender distribution. There are no data available on gender bias in the assessment of ligamentous structures using MRI images but a bias, even though unlikely, cannot be ruled out. Fourth, our results regarding the assessment of the SCR images could have been affected by the degree of knee flexion and rotation during MRI acquisition. The MPR technique described above is less affected by patient position since the reference planes are always aligned to the distal femoral anatomy.

## Conclusion

MPR images should be used when assessing the integrity of the ALL. The main advantage of this technique is that the assessment quality is independent of patient positioning during MRI acquisition and the ALL can be displayed in full length on one image.

## Abbreviations

MRI: Magnetic resonance imaging
MPR: Multi-planar reformation
SCR: Standard coronal reformation
ACL: Anterior cruciate ligament
ALL: Anterolateral ligament
ALC: Anterolateral complex
LET: Lateral extra-articular tenodesis
